# Thermal Niches of Two Invasive Genotypes of the Wheat Curl Mite *Aceria tosichella*: Congruence between Physiological and Geographical Distribution Data

**DOI:** 10.1371/journal.pone.0154600

**Published:** 2016-04-28

**Authors:** Lechosław Kuczyński, Brian G. Rector, Agnieszka Kiedrowicz, Mariusz Lewandowski, Wiktoria Szydło, Anna Skoracka

**Affiliations:** 1 Department of Avian Biology and Ecology, Institute of Environmental Biology, Faculty of Biology, Adam Mickiewicz University, Poznań, Poland; 2 United States Department of Agriculture (USDA-ARS), Great Basin Rangelands Research Unit, Reno, United States of America; 3 Department of Animal Taxonomy and Ecology, Institute of Environmental Biology, Faculty of Biology, Adam Mickiewicz University, Poznań, Poland; 4 Department of Applied Entomology, Faculty of Horticulture, Biotechnology and Landscape Architecture, Warsaw University of Life Sciences–SGGW, Warsaw, Poland; Nanjing Agricultural University, CHINA

## Abstract

The wheat curl mite (WCM), *Aceria tosichella* Keifer, is a major pest of cereals worldwide. It is also a complex of well-defined genetic lineages with divergent physiological traits, which has not been accounted for in applied contexts. The aims of the study were to model the thermal niches of the two most pestiferous WCM lineages, designated MT-1 and MT-8, and to assess the extent to which temperature determines the distribution of these lineages. WCM population dynamics were modeled based on thermal niche data from March to November on the area of Poland (>311,000 km^2^). The most suitable regions for population development were predicted and compared to empirical field abundance data. Congruence between modeled parameters and field data for mite presence were observed for both WCM lineages although congruence between modeled thermal suitability and mite field abundance was observed only for MT-8. Thermal niche data for MT-1 and MT-8 provide biological insights and aid monitoring and management of WCM and the plant viruses it vectors. The presented models accurately estimate distributions of WCM and can be incorporated into management strategies for both current and predicted climate scenarios.

## Introduction

*Aceria tosichella* Keifer (wheat curl mite, WCM) is among the most important eriophyoid mite pests of agriculture. The mite is distributed worldwide in cereal fields and grasslands infesting numerous species of domestic and wild grasses [1]. It causes direct plant damage by feeding on plant tissues. However, the primary economic impact caused by WCM is due to its ability to vector several destructive plant viruses (e.g. *Wheat streak mosaic virus*, *Wheat mosaic virus*, *Triticum mosaic virus*, *Brome streak mosaic virus*), thus complicating WCM and virus management efforts [1–3]. Due to the tiny size of WCM (ca. 200 μm) and its habit of sheltering within plant structures (e.g. leaf sheaths, grooves, beneath the palea or lemma within florets) the mite is often very difficult to detect and therefore has a high potential as an invasive species [1]. The spread of the WCM-virus system to cereal producing regions worldwide is of increasing importance. Although cereal growers and researchers have been aware of this mite-virus pathosystem for about seven decades [4] it continues to cause significant economic impact, both in long-established invasions, such as in North America [5], and more recently invaded regions like Australia and South America where it has become a new threat to winter cereal production [6, 7].

Despite more than 60 years of WCM management efforts [4], the need for more effective control strategies for this cereal pest is still urgent [1]. Efficient management may additionally be hindered by the fact that WCM is in fact a complex of cryptic species composed of several well-defined genetic lineages (‘genotypes’) [8–11]. Since the studies leading to this outcome are relatively recent, the presence of different genotypes or species within the WCM complex has not yet been accounted for in applied contexts. Such oversights may have serious consequences, as cryptic species or genotypes within species complexes may differ with respect to key biological traits, such as host range, life-history traits, tolerance to abiotic factors, insecticide resistance, and ability to transmit plant viruses [12–18]. WCM is a complex of at least eight genetic lineages, with host-acceptance traits ranging from polyphagous to highly host-specific [9, 19]. The global status of WCM as a pest of cereals is due mainly to two lineages, MT-1 and MT-8 (in USA reported as Type 1 and Type 2, respectively), that are highly polyphagous, invasive, and distributed worldwide [20]. Owing to the increased global trade of the host plants of these eriophyoid mites, as well as their wind-borne dispersal [1, 21] they pose a serious threat to cultivated cereals in any invaded area, especially where they encounter favourable agro-ecological conditions.

The occurrence and distribution of any organism is determined by various environmental factors, among which temperature is generally recognized as the most important [22–25]. For herbivorous insects and mites, changing temperatures directly influence development and fecundity, which combined determine population growth rates [26–28]. A thorough understanding of an organism thermal biology is of critical importance in applied contexts, since predictions of biological responses to temperature are used in population forecasting and management decision-making [29–31].

Moreover, changing climatic patterns may lead to changes in frequency of pest outbreaks their distributions [22, 29, 32, 33]. For example, in North America the recent trend towards warmer and longer autumns has resulted in an expanded time period for WCM to spread and increase populations on wheat as well as transmit viruses [1].

Several demographic parameters have been reported for WCM *sensu lato* under stable temperature regimes [1, 34]. However, growth responses over a wide range of temperatures and critical thermal thresholds have not yet been documented for this species nor for its respective genetic lineages. The goal of the present study was to establish empirical data for temperature-dependent population growth rates of the globally distributed WCM pest lineages MT-1 and MT-8. In addition, we assessed the extent to which temperature is the factor that determines the spatial distribution of the studied pests. First, using thermal physiological data derived from laboratory experiments, we estimated thermal niches of these pests by assessing the functional relationships between temperature and population growth rates. Second, on the basis of the thermal niche parameters, we modeled the spatial variation of potential population growth for these two genotypes on the area of Poland. Third, using randomly sampled field data from across Poland, we tested the congruence between the thermal niches and the occurrence and abundance of these pests.

## Material and Methods

### Ethic statement

Field permit: A portion of our field survey was conducted in the fields of the Danko Plant Breeders LTD with permission from the Director Zofia Banaszak.

### Mite stock colonies and mite genetic identification

Wheat was used as the host plant for stock colonies and experiments, since this cereal species is a common host for both studied genotypes and it is also a primary cereal crop in Poland and worldwide. Mite females used for experiments originated from stock colonies established by transferring field-collected female mites to potted wheat plants, variety Ostroga, and rearing through several generations. Females were distinguished from other stages on the basis of body dimensions. Females are much larger than males and immature stages (by at least 33%). Preliminary studies in which live WCM individuals were scored by size, with their sex subsequently confirmed by observation under a phase-contrast microscope, were conducted to validate this method of sexing. Mites originated from bread wheat, *Triticum aestivum* L. and triticale, x*Triticosecale* Wittm. plants that were collected from fields of the Danko Plant Breeders LTD located in Choryń (52°02'24"N, 16°46'09"E) and in Anteczków (51°50'53"N, 17°09'06"E). Stock colonies were established from individual mite specimens collected from two different field sites and host plants in Poland and they were subsequently identified as the two genetic lineages, MT-1 and MT-8 [19, 20]. Colonies were maintained in rearing cages in a growth chamber set at 23–25°C, photoperiod 16:8 (L:D) h, and ambient relative humidity.

Identification to species, i.e. *Aceria tosichella* species was based on morphological data [35] and identification of WCM lineages was based on molecular data. For morphological identification, specimens were mounted on slides and examined with a phase-contrast microscope. For molecular identifications the mitochondrial cytochrome c oxidase I (COI) gene sequence was utilized due to its efficiency in discrimination between cryptic species in the animal kingdom [36], as well as between genotypes within the WCM complex [19]. Molecular identifications were repeated on a regular basis through successive generations of the stock colonies. Specimens were soaked in ATL buffer to enable non-destructive DNA isolation [37]. Primers used for PCR were generic COI primers for eriophyoid mites, viz. bcdF01 and bcdR04 [38]. Subsequently, the PCR amplicon (650 bp) was sequenced with BigDye Terminator, version 3.1, in accordance with the manufacturer’s instructions, and products from sequencing reactions were analyzed on an ABI Prism 3130XL or 3730 Analyzer (Applied Biosystems). Trace files were checked and edited using MEGA6 [39], and a Basic Local Alignment Search Tool (BLAST) search of the sequence was performed on the National Center for Biotechnology Information (NCBI) GenBank database. GenBank accession numbers that represent MT-1 and MT-8 lineages are JF920077 and KC422635, respectively.

### Laboratory experimental design

Experiments were conducted from March 2013 to July 2015. Mites were reared in the laboratory stock colonies for at least ten generations before individuals were used in the experiments. Experiments were conducted at ten constant temperatures ranging from 1°C to 45°C, 60±5% relative humidity (the optimal humidity conditions for plant growth and WCM development according to preliminary observations) and 16:8 (L:D) in incubator chambers. From 15 to 20 females of each genotype were transferred from stock colonies to clean potted wheat plants (7–10 days old), using an eyelash glued to a preparatory needle. After 10 min, plants were checked to count the number of females that had successfully settled. This number was assumed to be the number of females engaged in the experiment (hereafter ‘experimental females’). Females that appeared to be killed or injured during transfer were removed from the plants during the post-transfer inspection. Each experiment (combination of mite genotype and each of the ten temperatures tested) was repeated six times. The total number of experimental females was 895 for WCM MT-1 and 898 for WCM MT-8. After 14 days, incubated plants were inspected and the number of mites was counted using a stereo-microscope. When necessary, plants were destroyed in order to ensure that all mites were found.

Additional experiments were made at temperatures below 0°C, viz. -5°C and -15°C. Due to technical constraints, these experiments were conducted in freezers, instead of incubator chambers. Plants from the stock colonies with the counted number of mites (10–15 per plant, six repetitions) were cut and located in the freezers. After 14 days the number of mites on thawed plants was counted using a stereo-microscope.

### Field study design

**Fig 1:**
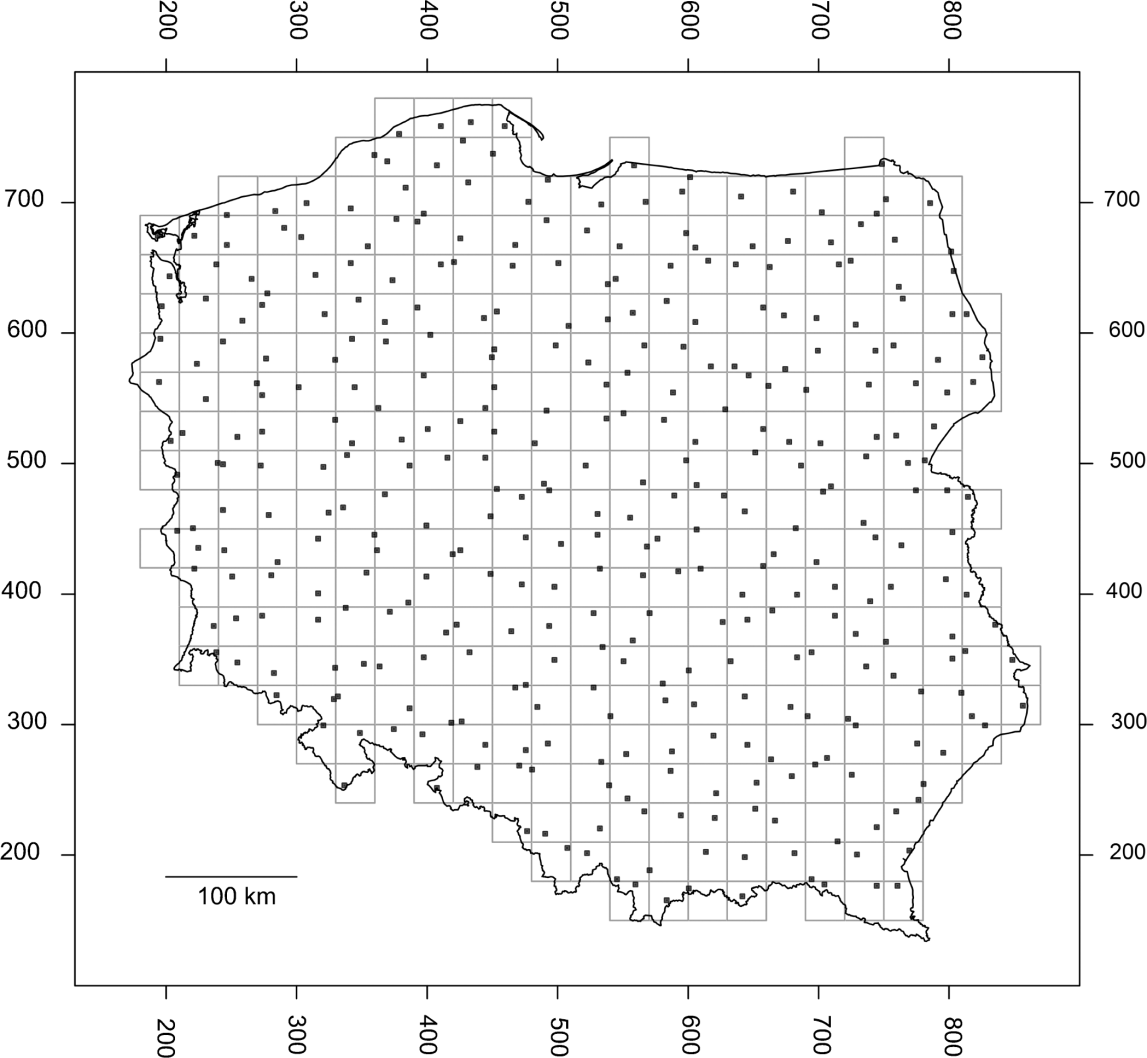
The area of Poland divided to 367 strata. Smaller squares were selected randomly within agrarian landscapes for plant sampling.

The field study was conducted on the area of Poland (311,888 km^2^) and included three summers of field collections (June-August, 2012–2014). To achieve an even distribution of sampling localities, a stratified random sampling scheme was used. The area of Poland was divided into 367 squares, each measuring 30x30 km, forming spatial strata. Within each stratum a 1x1 km square of agrarian landscape was selected at random (Fig 1). Randomisation was restricted to agrarian cover types based on the Corine Landcover database [40].

From the area within each 1 km^2^ square, cereals and wild grasses common to Poland were collected (at least one cereal species and two wild grass species), including bread wheat; triticale; rye, *Secale cereale* L.; oat, *Avena sativa* L.; barley, *Hordeum vulgare* L.; quackgrass, *Elymus repens* (L.) Gould; tall oat-grass, *Arrhenatherum elatius* (L.) Beauv. ex Presl & Presl; and smooth brome *Bromus inermis* Leyss. Plant samples were collected from all 367 sampling sites. Samples were replicated within a site: ten plants of each species were collected at random from each sampling area of approximately 100 m^2^. The localities accessed were not privately owned or protected. All plant species collected are common for farmland in Poland and are known hosts for various WCM genetic lineages [10, 19] and are not protected.

Samples were transported to the laboratory and subsequently examined for the presence of mites using a stereo microscope. The plant parts that were inspected were: leaves, leaf sheathes and inflorescences. Numbers of mite specimens were calculated on a per leaf, leaf sheath, or inflorescence basis. Specimens were prepared for DNA barcoding analysis as described above.

### Statistical analysis

For all computations R 3.2 was used [41].

#### Growth curves

An intrinsic population growth rate (*r*) was used as a measure of the reproductive performance of WCM genotypes under different temperature regimes. This was defined according to the formula:

$$r={\text{log}}_{2}\left(\frac{n}{n_{0}}+1\right)\;,$$ (1)

where: *n* represents the number of mites found 14 days after the start of an experiment and *n*_*0*_ represents the number of experimental females from the original transfer. The interpretation of this index is as follows: if *r = 1*, the population size did not change (i.e. no females reproduced but all survived, or the same number died as were born); if *r > 1* the population increased, indicating successful reproduction; if *r < 1* the population decreased (i.e. females did not reproduce or the reproduction rate was lower than the mortality rate). When the whole population was extinct (i.e. no specimens alive after 14 days) *r = 0*.

To assess the functional relationship between temperature and population growth rate [42] proposed the following model:

$$r(T)=\{\begin{array}{c} 0\Leftrightarrow T\leq T_{0}\\ aT(T-T_{0}){\left(T_{L}-T\right)}^{\frac{1}{m}}\Leftrightarrow T_{0}<T<T_{L}\\ 0\Leftrightarrow T\geq T_{L} \end{array}\;,$$ (2)

where *r* is the intrinsic population growth rate, *T* is the temperature in degrees Celsius, *T*_*0*_ is the low temperature developmental threshold, *T*_*L*_ is the lethal temperature (upper threshold) and *a* and *m* are parameters determining the shape of the estimated curve.

Because populations of WCM and their hosts did not survive in temperatures below the freezing point under laboratory conditions, this function was simplified by substituting *T*_*0*_ *= 0*, which reduces the Eq 2 to:

$$r(T)=\{\begin{array}{c} 0\Leftrightarrow T\leq 0\\ aT^{2}{\left(T_{L}-T\right)}^{\frac{1}{m}}\Leftrightarrow 0<T<T_{L}\\ 0\Leftrightarrow T\geq T_{L} \end{array}\;.$$ (3)

Parameters of Eq 3 (*T*_*L*_, *a*, *m*) were estimated using nonlinear least-squares [43].

#### Thermal niche modeling

Average monthly mean surface temperature were obtained from the WorldClim database http://www.worldclim.org [44]. These surface temperatures are available on a 30 arc-second resolution grid (which corresponds to approximately 1 km squares) and were generated using weather station records containing at least 10 years of data from the period 1950 to 2000. These surfaces were imported into R using the package raster [45], spatially queried to cover Poland only and re-projected to the spatial resolution of 1 km into the national projection “PUWG-1992” (EPSG 2180).

Laboratory experiments lasted for 14 days. In order to match the WorldClim data to this time interval, the mean monthly temperatures were linearly interpolated and the final data set comprised 26 layers containing reconstructed mean temperatures for 14 day periods.

#### Projection of population size

The model simulates population dynamics in 14-day intervals. Populations of both WCM lineages need at least 10–12°C to increase in numbers, so we started simulations at the time step beginning at the 29-th of April (according to the WorldClim data, the mean temperature for April is 8.1°C and for May 12.9°C) until the end of calendar year. The successive index of discrete 14-day periods considered in simulations is denoted hereafter as *t*.

Population size for each 1 km square at each time step *t* was calculated according to the formula:

$$N_{t}=N_{0}\prod_{t=1}^{n}\lambda_{t}\;,$$ (4)

where *N*_*t*_ is the population size at time *t*, *N*_*0*_ is the initial population size (set to 100 individuals in all simulations), *λ*_*t*_ is population finite rate of increase between time *t* and *t-1*, *n* is the number of time steps considered (which is the length of vector *t*).

By setting *λ = n/n*_*0*_ in Eq 1 we can derive the value of *λ* and substitute it into the Eq 4:

$$N_{t}=N_{0}\prod_{t=1}^{n}\left(2^{r(t)}-1\right)\;,$$ (5)

where *r(t)* is the intrinsic population growth rate estimated from Eq 3 using the mean temperature for the time period *t*.

The value of *N*_*t*_ cannot be treated as an absolute number representing population size. Instead, it should be interpreted as an index showing the population growth potential at a given temperature regime without any other constraints (in particular with no density-dependence). For this reason, we call the log-transformed *N*_*t*_ as the thermal niche suitability index (TNS) hereafter.

#### Model validation

For each sampling locality, the TNS was calculated using Eq 5 followed by the transformation TNS = ln(*N*_*t*_+1). The appropriate value of *t* was extracted from the sampling date by matching to a corresponding 14-day period.

The hurdle model [46] was used to validate the TNS index against the field data. This approach can separate habitat suitability into two components: abundance and occurrence. The abundance (or intensity, *sensu* Skoracka and Kuczyński [47]) is modeled as a truncated count process using Poisson or negative binomial distribution and is employed for positive counts only. The occurrence (or prevalence) is modeled as a binomial (presence/absence) process and is employed for zeros vs. larger counts. This approach accounts for systems in which the mechanisms that influence presence differ from those that influence abundance [48].

The hurdle model was fit using the glmmADMB package [49, 50], with the two parts of the model fitted separately. To allow for both zero-inflation and over-dispersion, the negative binomial distribution for modeling counts was used. To account for sampling effort, the logarithm of the number of stems in a sample was modeled as an offset.

## Results

### Population growth rates in relation to temperature

**Fig 2:**
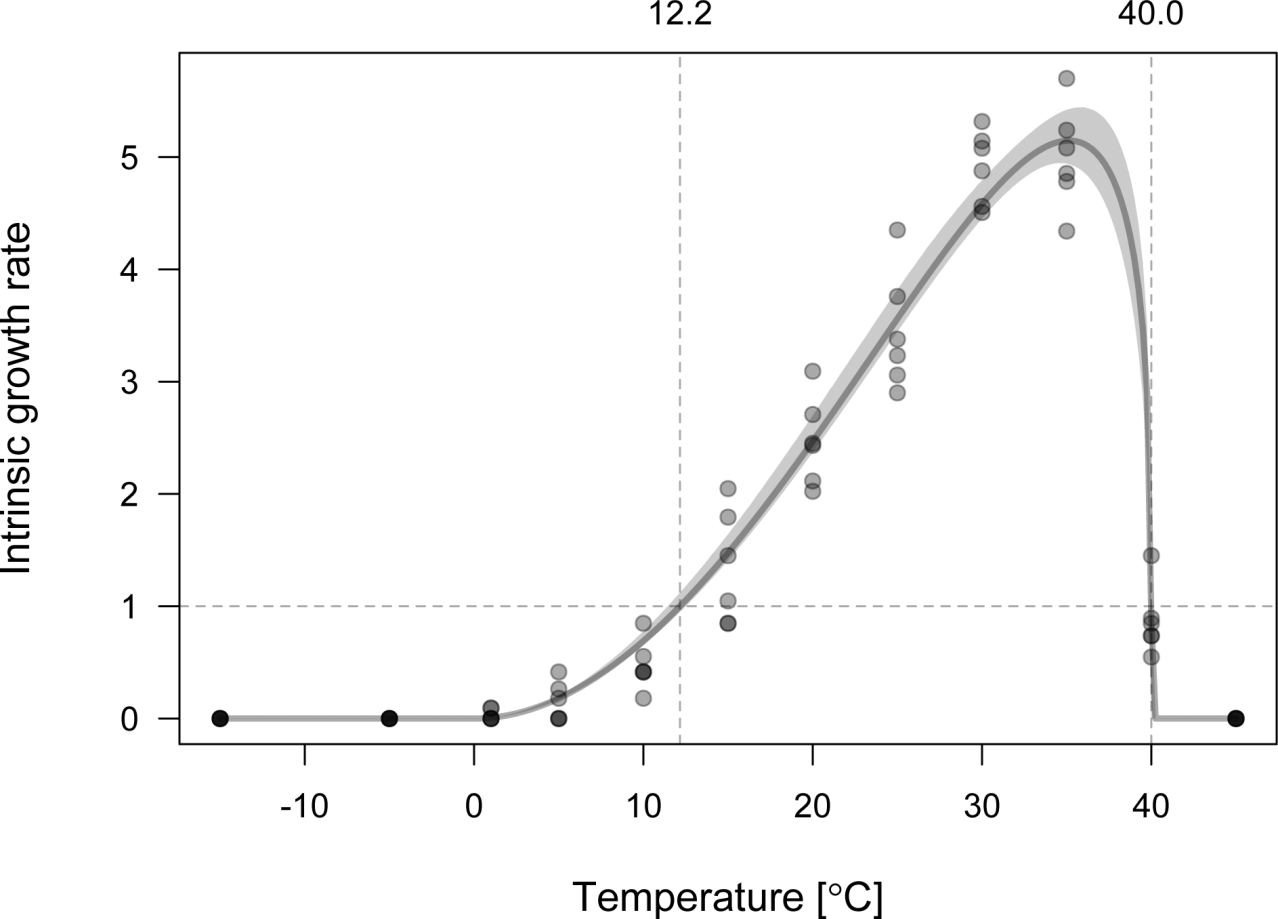
Relationship between temperature and population growth rate for *Aceria tosichella* lineage MT-1. Vertical dashed lines denote temperature ranges within which the population increases in numbers. Shaded region represents standard error band.

**Fig 3:**
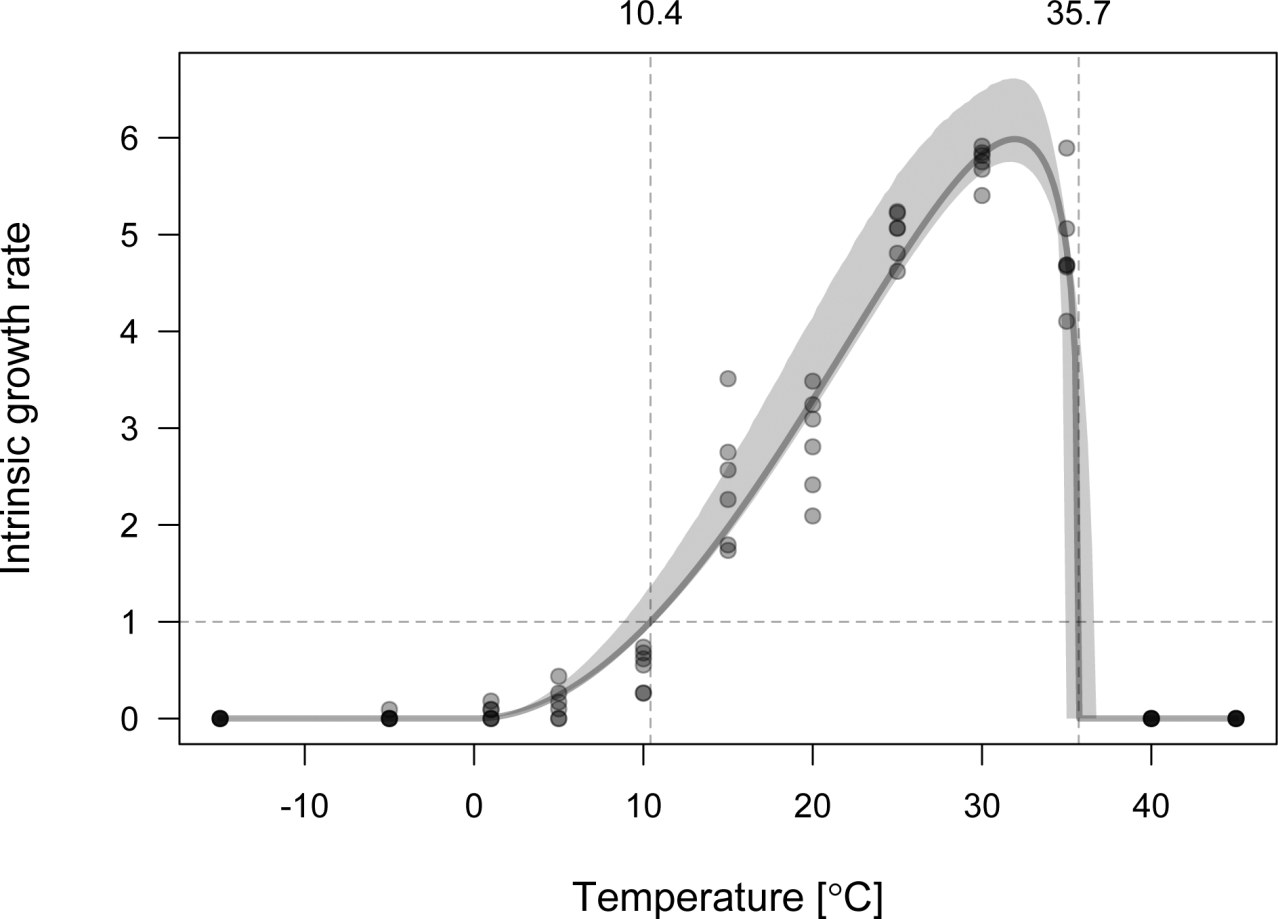
Relationship between the temperature and population growth rate for *Aceria tosichella* lineage MT-8. Vertical lines denote temperature ranges within which the population increases in numbers. Shaded region represents standard error band.

For both WCM lineages, with increasing temperature population growth rates increased slowly at lower temperatures, while at the highest temperatures growth rates showed a rapid decline (Figs 2 and 3). The optimal temperatures for population growth were 35.13°C and 31.93°C for MT-1 and MT-8, respectively. The temperature ranges within which populations were able to increase in numbers were: 12.2 to 40.0°C for MT-1 and 10.4 to 35.7°C for MT-8, with the highest temperatures representing the upper thresholds for survival (Table 1, Figs 2 and 3).

**Table 1:** Estimated parameters and standard errors (SE) of Briere growth curves (*a* and *m*) for *Aceria tosichella* lineages MT-1 and MT-8.

Parameter	MT-1		MT-8	
	Estimate	SE	Estimate	SE
*T*_*L*_	40.0	0.0032	35.7	0.4613
*a*	0.00269	0.00018	0.00428	0.00055
*m*	3.605	0.389	4.221	0.931

*T*_*L*_−maximal upper temperature threshold

### The spatiotemporal variation in intrinsic growth rate

**Fig 4:**
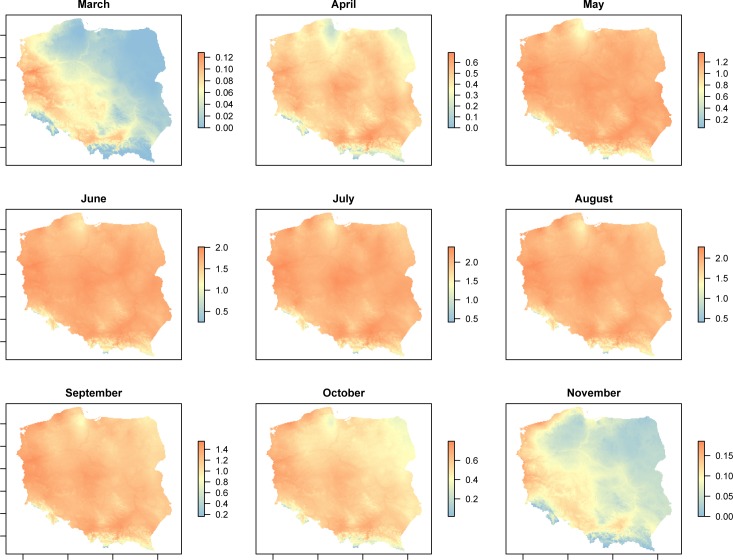
Spatial variation in intrinsic growth rate for *Aceria tosichella* lineage MT-1, predicted from fundamental thermal niche using mean monthly temperatures.

**Fig 5:**
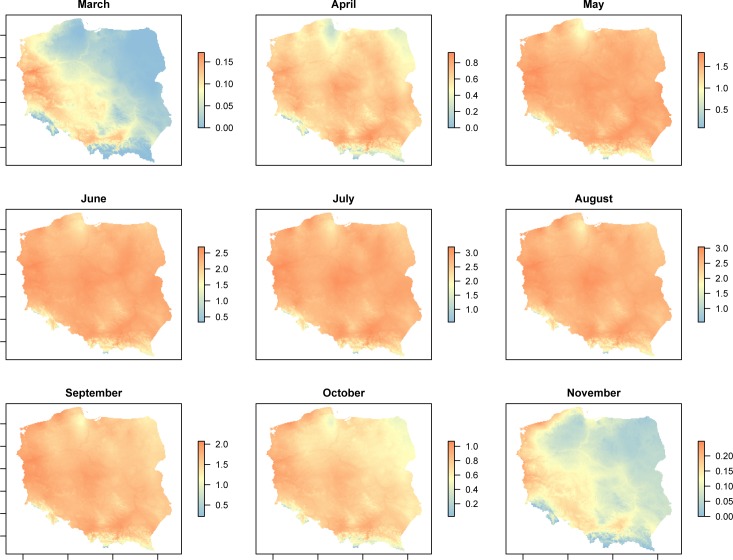
Spatial variation in intrinsic growth rate for *Aceria tosichella* lineage MT-8, predicted from fundamental thermal niche using mean monthly temperatures.

The spatiotemporal potential of intrinsic population growth rate in the area of Poland from March to November is presented for MT-1 and MT-8 in Figs 4 and 5, respectively. The greatest potential for MT-1 population increase (i.e. r>1) is from May to September, whereas for MT-8 it is from May to October. The southwestern half of Poland offers suitable thermal conditions for the persistence of both lineages in early Spring (March) and late Autumn (November), with the west-central region and northwestern coastline being the most favorable (Figs 4 and 5).

### Model validation against field data

**Fig 6:**
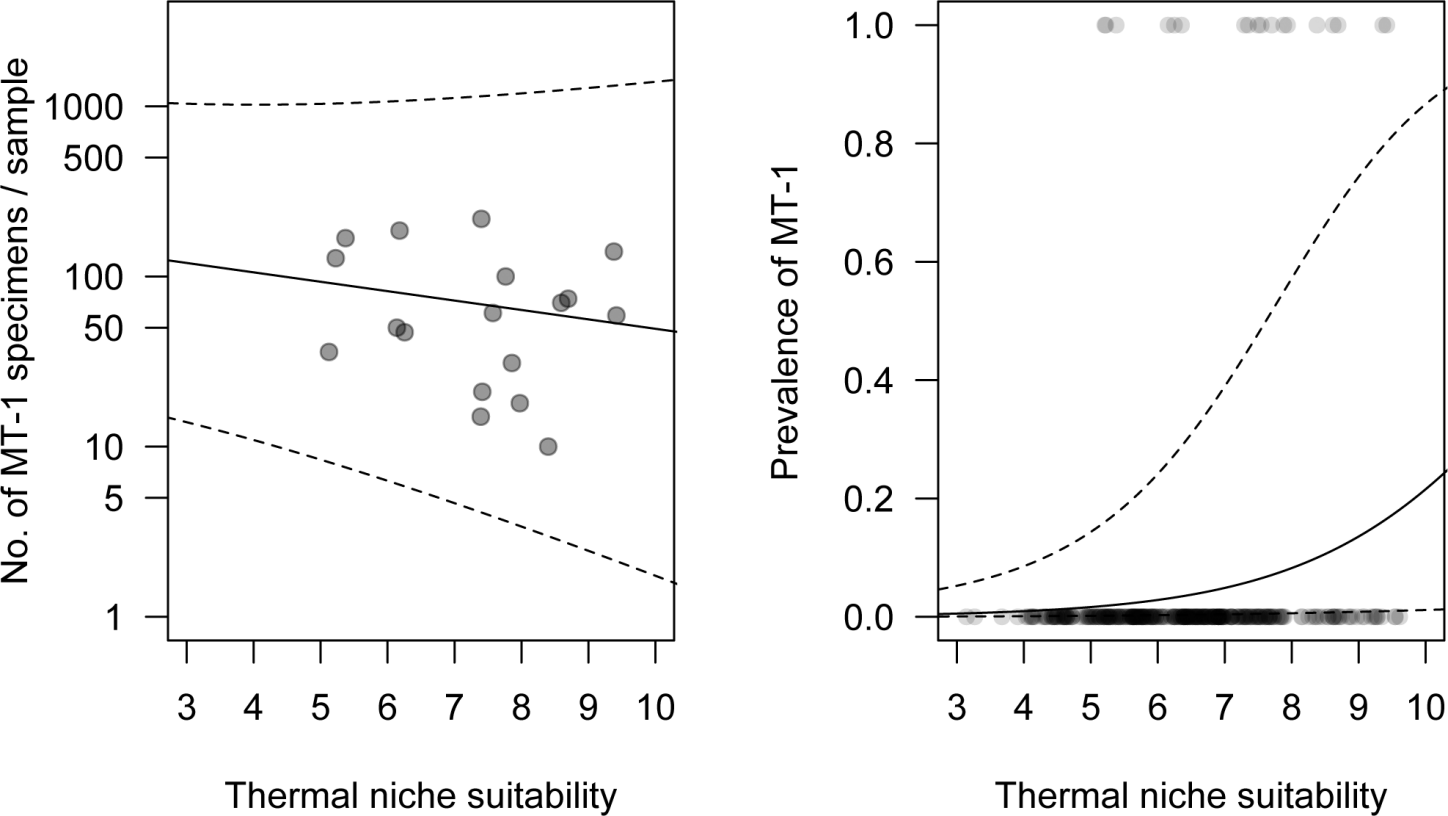
**The hurdle model relating thermal niche suitability (TNS) to abundance (A) and prevalence (B) of *Aceria tosichella* lineage MT-1.** Solid lines are predictions from the model and dashed lines represent standard error bands. Abundance of MT-1 in a sample was not dependent on TNS, while prevalence was.

The prevalence of MT-1 in Poland depended significantly on thermal niche suitability, whereas MT-1 abundance was not related to thermal suitability. The more suitable the thermal niche, the more frequently MT-1 specimens were found, but suitable thermal conditions did not influence mite numbers (Table 2, Fig 6).

**Table 2:** Parameter estimates of the hurdle model for *Aceria tosichella* lineage MT-1 relating the observed abundance (truncated negative binomial distribution with log link) and prevalence (binomial distribution with logit link) to thermal niche suitability (TNS).

Parameter	Estimate	SE	z-value	p	Estimate	SE	z-value	p
	Count model				Zero hurdle model			
Intercept	1.74	1.02	1.70	0.089	-10.32	1.40	-7.38	<0.001
TNS	-0.13	0.14	-0.93	0.351	0.56	0.19	2.95	0.003
Dispersion	1.48	0.49						

**Fig 7:**
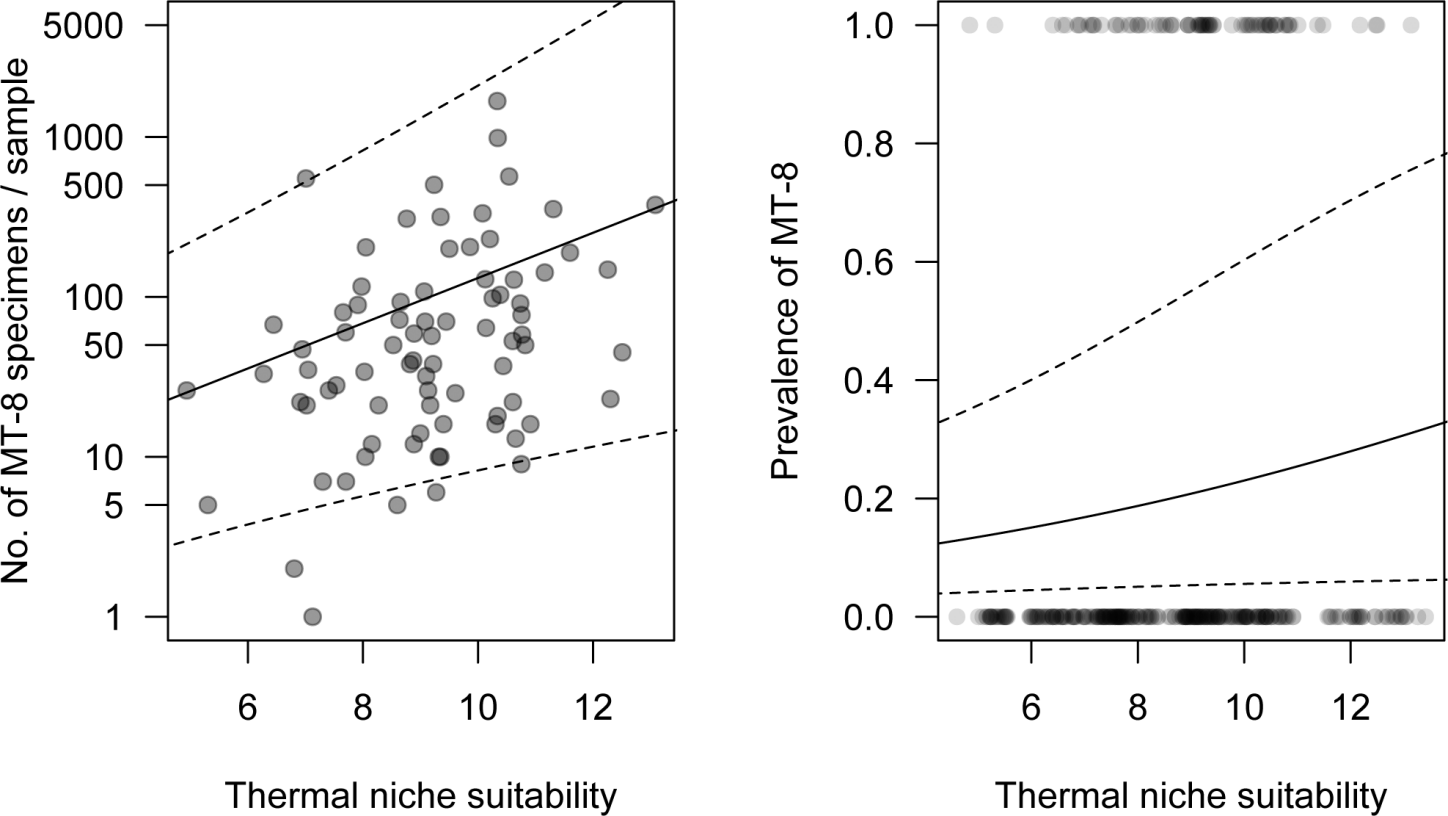
**The hurdle model relating thermal niche suitability (TNS) to abundance (A) and prevalence (B) of *Aceria tosichella* lineage MT-8.** Solid lines are predictions from the model and dashed lines represent standard error bands. Both abundance and prevalence of MT-8 were significantly dependent on TNS.

Both the prevalence and abundance of MT-8 in Poland significantly and positively depended on thermal niche suitability. The more suitable the thermal niche, the more frequently MT-8 specimens were found and in higher numbers (Table 3, Fig 7).

**Table 3:** Parameter estimates of the hurdle model for *Aceria tosichella* lineage MT-8 relating the observed abundance (truncated negative binomial distribution with log link) and prevalence (binomial distribution with logit link) to thermal niche suitability (TNS).

Parameter	Estimate	SE	z-value	p	Estimate	SE	z-value	p
	Count model				Zero hurdle model			
Intercept	-1.81	0.96	-1.88	0.060	-5.94	0.61	-9.74	<0.001
TNS	0.32	0.10	3.14	0.002	0.13	0.07	1.98	0.048
Dispersion	0.50	0.10						

## Discussion

In this study we provide for the first time empirical data on temperature-dependent population growth of the two most pestiferous, widespread and invasive wheat curl mite (WCM) lineages, viz. MT-1 and MT-8. Through virus transmission these mites cause yield losses up to 83% in wheat, representing millions of dollars of lost revenue each year [1, 51, 52]. No information on thermal niches of these cereal pests has been available until now, although such knowledge is critical to the development of effective area-wide management strategies. In the past, some life-history parameters (e.g. egg development, mite survival) have been observed under different temperatures, however growth responses over a wide range of temperatures have not been provided to date [1]. Moreover, none of the previous studies considered the existence of different genetic lineages within WCM. Physiological thermal niches of these WCM lineages tested in this study, differed, with MT-1 performing better at higher temperatures compared to MT-8. Also Schiffer et al. [53] found that two WCM biotypes in Australia differ in their tolerance to regional climates. However, since the Australian lineages were identified on the basis of different DNA markers, we cannot be sure if they correspond to MT-1 and MT-8 lineages without making direct comparisons. The physiological differences between lineages found in this study support previously reported evidence (viz. DNA marker differences [19]) showing that MT-1 and MT-8 are distinct biological entities (perhaps separate species). Thus cereal protection strategies should account for each lineage separately, especially with regard to local climatic conditions. Wosula et al. [54] have also shown that these lineages differ in virus transmission, underscoring the importance of effective discrimination between lineages.

In addition to differential virus transmission [54] and thermal niches, WCM lineages likely differ in other biological traits that may affect their pest status and potential management tactics; these could include tolerance to other abiotic or biotic factors, life-history parameters, and dispersal propensity. Such biological data is unavailable for WCM lineages and further experimental work is necessary to fill these gaps. Data generated in this study on thermal ranges and thermal optima for MT-1 and MT-8 will be useful in establishing optimized rearing conditions for further experimentation focusing on the biology and ecology of WCM lineages, as well as for efficiently rearing large numbers of mites in order to produce sufficient DNA for genome sequencing and transcriptomic studies of traits that differ among WCM lineages.

Optimal temperature conditions under which the MT-1 and MT-8 lineages attained the highest population growth were higher (>35°C and >31°C, respectively) than those of other eriophyoid pests for which data is available, e.g. tomato russet mite, dry bulb mite, citrus rust mite [26, 55, 56]. This high thermal tolerance of MT-1 and MT-8 suggests that their expansion into many new areas is likely to be favored by climate warming, as has been reported for other pest species and associated pathogens [32 57, 58]. Moreover, if climate warming alters WCM presence and abundance it would consequently increase the risk of virus transmission by WCM. Indeed, in Australia warm temperatures have been shown to support large WCM populations, thereby intensifying the spread of WSMV in cereal crops [7]. It has also been suggested that climate change is one of the factors responsible for recent spread of WSMV in central Europe via WCM [59].

Thermal tolerances of species are often important factors in determining their distributional ranges, thus the characterization of thermal limits is essential for predicting a species ability to colonize a new area [60, 61]. Thus, thermal niche data is frequently adopted to improve models that attempt to predict such colonization by pest or invasive species and to support the development of control strategies [62–64]. In this study, thermal niche data for the MT-1 and MT-8 lineages of WCM, assessed over a wide range of temperatures, were used to model the temperature-dependent spatiotemporal potential distributions and abundances of these lineages within the area of Poland. The predicted population dynamics from March to November were similar for both the MT-1 and MT-8 lineages with the majority of the country offering excellent conditions for growth of both lineages from May to September. This five-month period of WCM population development should be sufficient for virus transmission by WCM [1]. Additionally, the warmest region of Poland, the southwest, offers favorable thermal conditions for MT-1 and MT-8 population growth in March, October and November. Taking into account scenarios of climate change in Poland during the 21st century that predict temperature increases across the country [65], it is likely that more of the country will be suitable for MT-1 and MT-8 development in early Spring and late Autumn and perhaps the warmest parts of the country will become suitable for WCM development also in Winter (December, January, February). For this reason, the presence of MT-1 and MT-8 in Poland and other European countries should be closely monitored. The population models provided in this study, incorporating parameters of thermal requirements, represent a useful tool for understanding MT-1 and MT-8 population dynamics and for evaluating efficacy of further successful WCM management practices.

Ecological niche models predict areas of species occurrence and may help to elucidate the factors that affect their distributions [66–68]. However such models are rarely field-checked and evaluated [69] due to the inherent logistical challenges. This is of concern especially for pests invading diverse regions. In this study, predictive distribution models developed for the WCM lineages MT-1 and MT-8 lineages were tested through a comprehensive field survey of extensive and representative sampling over the entire area of Poland. There was a strong correspondence between modeled thermal niche suitability and field prevalence data for both the MT-1 and MT-8 lineages. However, the congruence between modeled thermal suitability and mite field abundance was apparent only for MT-8, whereas field abundance of MT-1 was not correlated with modeled thermal suitability. Thus, both WCM lineages occur in areas that are thermally suitable for them, whereas there is a difference in the extent to which the two studied lineages thrive in these areas. Based on the model, field prevalence of WCM lineages is more directly connected to temperature conditions, whereas WCM field abundance may be associated with other factors that have yet to be investigated, to which MT-1 and MT-8 lineages respond differently. Our preliminary (data not shown) observations suggest that competition between different WCM lineages may play an important role in their relative field abundance. Thus, it could be hypothesized that between these two invasive WCM lineages that share some host plants, MT-1 is a lesser competitor compared to MT-8. Thus, in order to avoid competition with MT-8, MT-1 mites may opt for wind dispersal more frequently, resulting in increased mortality due reduced off-host survival of WCM [52]. This hypothesis could be experimentally tested and could have important management ramifications in areas where both lineages are invasive. Notwithstanding, our models successfully predicted areas in which MT-1 and MT-8 lineages occur over an area of more than 311,000 km^2^, thus showing the risk areas for these lineages that should be included in monitoring for these important pests of cereals.

## Conclusions

This study determined for the first time the thermal requirements for effective population growth of two the most invasive WCM lineages, MT-1 and MT-8, an important step in understanding how environmental factors affect population dynamics and invasiveness of these pests. Ascertaining temperature-dependent growth responses of MT-1 and MT-8 provides insights into their biology and for monitoring and management of WCM. With advancing global climate change, increased knowledge of thermal niches of invasive pest populations is crucial to predicting the future distributions of invasive pest populations in light of predicted climate warming. Improved understanding of growth parameters under different temperature regimes is also critical to mass rearing applications for both basic and applied experimental contexts. A foundational strategy of WCM management is to predict its potential distribution on cereals and alternative “green bridge” hosts for both the mites and the viruses they transmit [70]. The models developed in this study include data on the thermal niches of the MT-1 and MT-8 lineages and could be incorporated into management strategies based on the modeling of potential distribution of these lineages and their transmitted viruses in regions where cereals are grown.

Assessing pest prevalence and abundance in the field is also fundamental to the design of pest management strategy and tactics, and whenever possible should be incorporated to the evaluation of species distribution or niche models. Assessment of infestation parameters, based on extensive field sampling on the whole area of Poland, showed the congruence between thermally suitable areas for MT-1 and MT-8 lineages and their occurrence in the field, validating these models as tools for explaining the relationship between temperature and the frequency of WCM lineages, as well as for prediction of outbreaks of these agricultural pests and optimization of control strategies.
